# Understanding the use of digital technologies to provide disability services remotely during the COVID-19 pandemic; a multiple case study design

**DOI:** 10.1186/s12913-024-10652-6

**Published:** 2024-03-11

**Authors:** Jennifer Fortune, Manjula Manikandan, Sarah Harrington, Owen Hensey, Claire Kerr, Sebastian Koppe, Thilo Kroll, Grace Lavelle, Siobhán Long, Malcolm MacLachlan, Denis Nolan, Meriel Norris, Jason O’Reilly, Mary Owens, Aisling Walsh, Michael Walsh, Jennifer M. Ryan

**Affiliations:** 1 School of Population Health, RCSI University of Medicine and Health Sciences, Dublin, Ireland; 2 Public and Patient Involvement (PPI) Partner, Cork, Ireland; 3 Central Remedial Clinic, Dublin, Ireland; 4 School of Nursing and Midwifery, Queen’s University Belfast, Belfast, UK; 5 Enable Ireland, Galway, Ireland; 6 School of Nursing, Midwifery and Health Systems, UCD IRIS, University College Dublin, Dublin, Ireland; 7 Institute of Psychiatry, Psychology & Neuroscience, King’s College London, London, UK; 8 Enable Ireland, Dublin, Ireland; 9 National Clinical Programme for People with Disability, Health Service Executive, Dublin, Ireland; 10 Assisting Living & Learning Institute and Department of Psychology, Maynooth University, Kildare, Ireland; 11 NCBI, Dublin, Ireland; 12 College of Health, Medicine and Life Sciences, Brunel University London, London, Uxbridge UB83PH UK

**Keywords:** Implementation, Qualitative methods, Disability, Technology, Telehealth

## Abstract

**Background:**

Using digital technologies to provide services and supports remotely may improve efficiency and accessibility of healthcare, and support people with disabilities to live independently. This study aimed to explore the experience of using digital technologies to access and provide disability services and supports during the Covid-19 pandemic, from the perspective of people with disabilities, families and service providers.

**Methods:**

Using a multiple case study design, we purposively sampled three cases based on service user characteristics and geographical reach of the service. We conducted semi-structured interviews with 40 service users and service providers. Topic guides and analysis were informed by the Consolidated Framework for Implementation Research (CFIR). Analysis followed a largely deductive approach, using the CFIR constructs as a coding framework. A summary memo was developed for each case. Influence and strength of each construct was rated to identify constructs that influenced implementation of digital technologies. Ratings were compared across services to identify facilitators and barriers to implementation.

**Results:**

Service users and providers were positive about using digital technologies to access and provide disability services and supports remotely. Advantages over in-person delivery included reduced travel time, increased opportunity for peer support and peer learning, more choice and opportunity to participate in activities, and an enhanced sense of self while accessing services from the secure environment of their home. The urgency to identify new modes of service delivery to meet the needs of service users during Covid-19 was a strong facilitator but did not necessarily result in successful implementation. Other factors that were strong facilitators were the use of adaptations to enable service users to access the online service, service users’ willingness to try the online service, service users’ persistence when they encountered challenges, and the significant time and effort that service providers made to support service users to participate in the online service. Barriers to implementation included the complexity of accessing online platforms, poor design quality of online platforms, and organisations prioritising in-person delivery over online services.

**Conclusions:**

These findings may allow service providers to leverage facilitators that support implementation of online disability services and supports.

**Supplementary Information:**

The online version contains supplementary material available at 10.1186/s12913-024-10652-6.

## Background

In Ireland, 13.5% of the population of children and adults reported having a disability in the 2016 census of the population [1]. Approximately 7% of people under 20 years reported having a disability and this increased with age to 10% in those aged 40 years and 20% in those aged 60 years [1]. People with disabilities often experience reduced participation in employment and daily activities, and poorer health than those without disabilities [2–4]. They also face more barriers to accessing services and supports, which are critical for enabling them to live healthy and independent lives [5]. Access to services and supports is mandated by the United Nations Convention on the Rights of Persons with Disabilities (UNCRPD) [6]. Measures to prevent the spread of COVID-19 intensified barriers for people with disabilities in accessing services and supports [7–9]. Consequently, the COVID-19 pandemic disproportionately impacted people with disabilities and deepened pre-existing health inequalities [8, 10, 11].

In an attempt to reduce the impact of the COVID-19 pandemic on people with disabilities, many service providers used digital technologies to deliver services and supports remotely. Although many service providers and service users value in-person services, using digital technologies to provide services remotely may improve efficiency, accessibility and quality of healthcare [12]. A recent review of telemedicine for people with neurological conditions such as stroke and multiple sclerosis found that telemedicine improves motor function, symptoms of depression and quality of life compared to usual care [13]. For people with disabilities, the benefits of using technology to deliver services and supports remotely go beyond health. Technology may also be used to provide services and supports that promote participation in everyday life [14].

The use of digital technologies during COVID-19 highlighted its potential to provide services and supports to people with disability in Ireland. However, embedding technologies within routine practice often depends on context specific interactions and local contingencies [15]. Using technology to provide disability services may present specific challenges. The National Clinical Programme for People with Disability (NCPPD) in Ireland surveyed disability service providers to understand how technology was used to remotely provide services and supports to people with disability during COVID-19 [16]. Service providers reported several benefits. These included high levels of engagement among some service users, being able to assess service users in their home environment, improving access to services, and more efficient use of service providers’ and service users’ time and resources. Challenges included lack of appropriate equipment and training, poor internet connectivity, accessibility difficulties for people with certain impairments such as intellectual disability and hearing impairment, and dependency on family members. Service providers reported that some service users struggled to view interpreters and it was difficult to discuss sensitive issues on a video call.

A review describing how services were provided to people with disabilities during COVID-19 identified similar benefits and barriers to providing services remotely [17]. The review also highlighted that using technology may increase staff workload and increase reliance on informal caregivers, which may lead to burnout. However, few studies used qualitative methods to identify the barriers and facilitators to using technology to provide disability services remotely. None explored the perspectives of people with disabilities [17]. An understanding of the barriers and facilitators to implementation from the service user’s perspective is necessary to enable service providers to effectively provide services and supports. It is also a critical element within the context of the UNCRPD and its’ associated ethos of “nothing about us, without us”.

This study aimed to explore the experience of using synchronous digital technologies to access and provide disability services and supports in Ireland during the COVID-19 pandemic, from the perspective of people with disabilities, their families and service providers.

## Methods

We used a multiple case study design to address the aim of this study. The case study approach is particularly useful for generating an understanding of a complex issue in its real-life context [18]. Studying multiple case studies simultaneously allowed us to make comparisons across case studies and obtain a broader understanding of digital technology use for providing and accessing disability services and supports. We defined cases as services or components of a service in which service users and service providers interacted for a specific purpose (e.g., assessment, intervention, support group). Eligible services included medical and allied health professional services, assistive technology services, and support services.

### Sampling

We purposively sampled five cases through the NCPPD and other clinical gatekeepers working in disability services, according to the following criteria: (1) characteristics of individuals eligible for the service in terms of disability type and age (child or adult); (2) characteristics of the service in terms of the number of service users who attended the service and the format of the service (group or one-to-one); (3) geographical reach of the service (local or national) and if local, the location of the people who attended the service (rural or urban). Sufficient and timely access to potential participants was also a criterion for selecting cases. The goal of purposive sampling in this project was to obtain a varied sample of services that allowed sufficient depth of inquiry to generate evidence that informs implementation of synchronous digital technologies in disability services. We were unable to pursue evaluation work at two of the five cases because of difficulties obtaining approval and access to participants within the study’s timeline.

Within each of the remaining three cases, we purposively sampled key informants to participate in interviews. Key informants were service users, i.e., people with a physical, intellectual and/or sensory disability (aged 8 years and above) who used the service or support, and service providers, i.e., any person involved in implementation of digital technologies including health professionals, volunteers, managers, and IT staff. Where people required support to access services, we also interviewed those who supported them such as family members. Factors considered when sampling service users were: age, level of engagement with accessing the services using digital technology, use of assistive technology, level of support required to access services and geography. We purposively sampled service providers based on their role. We aimed to interview approximately 15 key informants in each case, with proportionately more service users than service providers interviewed. The number of interviews conducted was partly determined by the scale of digital technology use in each case.

An individual at each service, who was known to service users, acted as a gatekeeper for the case by providing potential participants with information about the study on behalf of the research team. To maximise participation, study documentation was provided in alternative formats as required. Ethical approval was obtained from RCSI’s Research Ethics Committee (reference: 202101018) and Enable Ireland’s Research Ethics Committee (reference: RA77). All participants over 18 provided consent. Participants under 18 years provided assent and a parent provided consent.

### Settings

The three cases are described in Table 1. Case A was virtual technology clubs. The purpose of the clubs was the learn about and discuss the latest technology, equipment and apps that can help overcome any barriers in the daily lives of people with sight loss. Seven regional clubs were developed, which were each facilitated by at least one technology trainer. Case B was named by the organisation as the “Virtual Service”. This purpose of the service was to replicate the in-person day services provided by the organisation across multiple locations. Service users were adults. Service users could join a chat room stream to meet and talk and attend structured activities on a separate stream. Activities were provided by staff members, volunteers and third-party contractors, and included Yoga, bingo, cooking, art, quizzes and self-advocacy training. The service was developed at two centres before becoming a national service. Case C was paediatric therapy groups for children aged 8-17 years. The groups were delivered in one centre and were typically facilitated by two clinicians.

**Table 1:** Description of the three included cases and data collection

**Case identifier and title**	**Description of organisation**	**Description of service**	**Data source**
Case A; Virtual Technology Clubs	National sight loss agency	**Scope:** The purpose was to learn about and discuss the latest in technology, equipment and apps that can help overcome any barriers in the daily lives of people with sight loss. **Stakeholders:** Service users were adults (≥18 years). Each club was facilitated by at least one Technology Trainer. The Technology Trainer is a role that existed in the organisation before the development of the clubs. These trainers initially had more of a didactic role, where they imparted information, but took on a more faciliatory role as the clubs developed and service users learned from their peers. **Platform:** Service users joined the club using Microsoft Teams. They were either sent a link to join on their computer or smart device, or were given a phone number to dial into meetings (audio only). **Location and frequency:** Seven regional clubs were developed across Ireland during a period of Covid-19 restrictions in 2020. The clubs initially met once per week. Out of the seven clubs, two clubs now meet once every 2 weeks. The remaining five clubs continue to meet once per week.	13 interviews; 2 technology trainers; 11 service users. 3 service users chose to join clubs using the phone (audio only). Service users and service providers from three clubs (Dublin, West of Ireland, and South-East of Ireland). Data collection November 2021.
Case B; “Virtual Service”	National organisation that provides services to children and adults with disabilities and their families in 15 counties.	**Scope:** The purpose was to replicate the in-person day services provided by the organisation across multiple locations. A chat room stream, for service owners to meet and talk, and a separate structured activity stream were developed. The virtual service provided a range of services to adult service owners. A menu of online classes was developed (Yoga, Zumba, Theatre, Bingo, Cooking, Art, Quizzes, Audio Book Club, music, creative writing, meditation, travel news, entertainment news, pet’s corner, outdoor walks, concerts, plays) resulting in a range of activities being available for adults to access remotely. Activities were developed in partnership with service owners and the service remained open to their suggestions throughout the development process. The content of activities was subsequently adapted to include self-advocacy, mental health and well-being and other educational/training content. **Stakeholders:** Service owners were adults (≥18 years). Activities were provided by staff members, volunteers and third-party contractors. A new “virtual support worker” role was developed, which service owners applied for and were subsequently trained to facilitate and lead activities. They received additional train-the-trainer and safeguarding training to support them in their roles. The virtual service was supported by an external partner that provided project management support, volunteers, training support, tech support, funding, and devices. **Platform:** Service owners joined using Microsoft Teams. **Location and frequency:** The virtual service was developed at two centres during a period of Covid-19 restrictions. It was replicated in other centres and subsequently became a National Virtual Service. It was originally provided five days per week from 9am to 5pm with six activities scheduled per day. It is now delivered in a blended format. between 10 am and 3 pm, with activities scheduled between 11 am and 3pm that service owners can access online or face-to-face in centres.	16 interviews; 9 service owners, 2 support people, 5 service providers. Service owners and service providers from three regional centres (Dublin, Limerick and Kerry). Data collection February to July 2022.
Case C; Paediatric therapy groups	National organisation that provides services to children and adults with disabilities and their families in 15 counties.	**Scope:** The purpose was to provide virtual group services to children. Groups included a virtual quiz, a virtual craft group, music group, social hang out group and footie talk group. **Stakeholders:** Participants in the group ranged in age from 8-17 years. Content for each group was led by a therapist with service user input and suggestions. For the virtual craft group, participants received craft supplies in the post in advance of the group. Groups were typically facilitated by two clinicians. The children were matched to a group according to the age range that was thought appropriate to manage by facilitators. Some groups were delivered by an external provider (e.g. craft and music group). Health professionals employed by the organisation supported the sessions by managing the technical aspects and group dynamics. As only 9 people could be viewed on screen at once, group size was restricted to 6 service users to allow up to three staff members support the session. **Platform:** Service users joined using Microsoft Teams. **Location and frequency:** The groups were developed at one centre during a period of Covid-19 restrictions. Some groups occurred in a once weekly, six-week block. Other groups occurred once a month or at the request of service users. The service is no longer being delivered.	11 interviews; 3 service users; 3 support people; 5 service providers. Service users and providers from one regional centre in West of Ireland. Data collection July to September 2022.

We will hereafter refer to each case as an “online service”. The online services were provided by two national organisations. The individual data collection periods varied by site (Table 1); all data were collected between November 2021 and September 2022.

### Data collection

We conducted in-depth semi-structured interviews with key informants. Participants were given the choice to conduct interviews by telephone or video-call. Topic guides were developed collaboratively by service providers, researchers and people with disabilities and informed by the Consolidated Framework for Implementation Research (CFIR) [19], the Framework for Defining User Engagement with Technology [20], and the Telehealth Usability Questionnaire [21]. Different topic guides were developed for each key informant group, i.e., person with disability, support person and service provider. Topic guides were further adapted depending on the age of the person with disability and the role of service providers. Topic guides are provided in Additional file 1. Adaptations were made within interviews to maximise participation, for example by providing topic guides in advance in written form if preferred by the participant. Interviews were audio-recorded and transcribed verbatim.

### Data analysis

Transcripts were pseudonymised and imported into data management software (NVivo V.12: QSR International). We used the CFIR to support us to systematically identify factors that influence implementation and produce findings that inform stakeholders how to improve implementation. The CFIR provides a menu of 39 constructs that have been associated with effective implementation arranged across 5 domains.

**Fig. 1:**
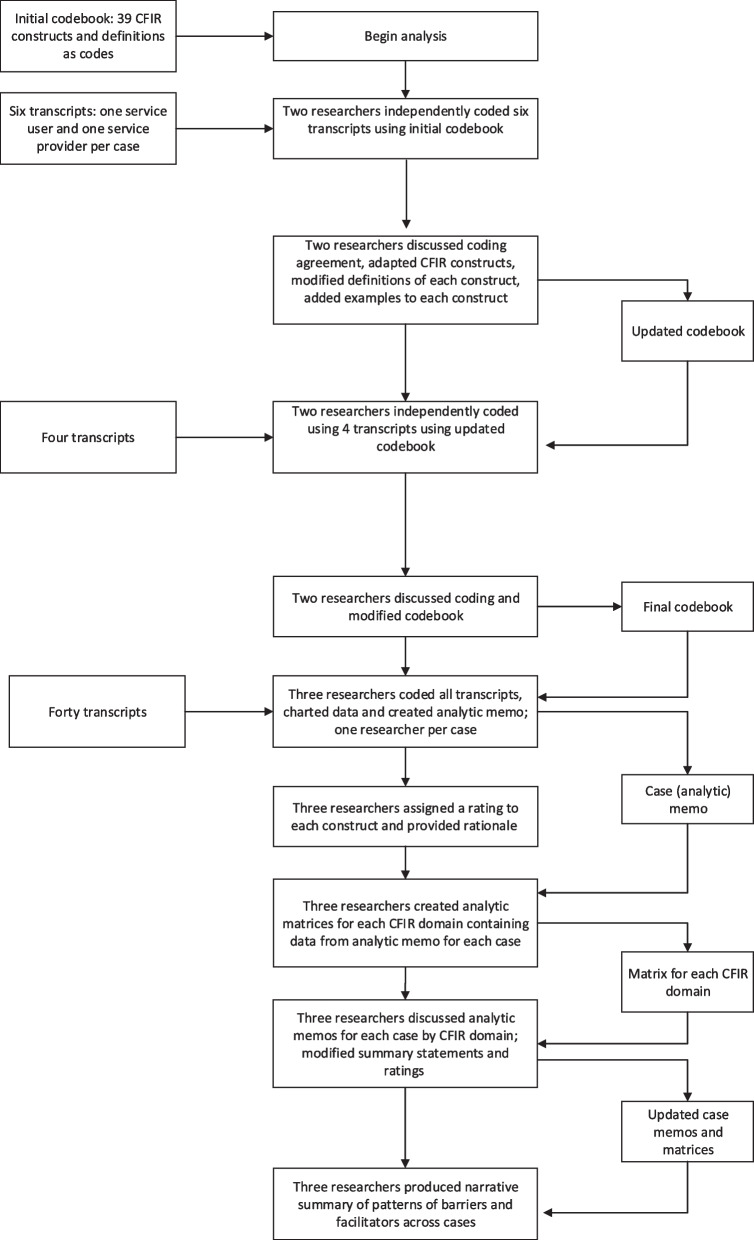
Analytic process

We used the CFIR to analyse the data using a similar approach to that described by previous studies [22, 23]. The process for analysis is summarised in Fig. 1. We developed an initial codebook that included all 39 CFIR constructs and their definitions as codes. Two researchers independently coded six transcripts using this initial codebook; one service user and one provider transcript from each online service. One researcher merged the two coding files to assess level of agreement. The two researchers met to discuss similarities and differences in codes used for each segment. Where codes differed, the researchers discussed and agreed on the code to use. During this process, we adapted the existing CFIR constructs according to our data. Specifically, we separated the following six constructs into service user and service provider components: relative advantage, knowledge and beliefs about the innovation, self-efficacy, individual stage of change, individual identification with the organisation, other personal attributes. During this process the researchers also made minor modifications to CFIR construct definitions to use the language found in our data and created examples of what to include under each code.

The two researchers subsequently coded an additional four transcripts independently. These files were also merged and the researchers met again to assess the level of coding agreement, discuss any discrepancies, and make minor changes to the codebook to ensure consistency between researchers. Following this discussion, a final codebook was produced. Three researchers independently coded the transcripts using the final codebook; one researcher per case. Constructs without data coded to them were labelled as “missing”. Missing indicated that interviewees were not asked about the presence or influence of the construct, or if asked about the construct their response was coded to another construct. Four transcripts (10%) were coded using the final codebook by all three researchers and discussed to ensure consistency in coding.

Following coding, the researchers charted data according to CFIR constructs and produced an analytic memo for their respective case organised by CFIR construct. Each memo included a summary statement for the construct with supporting data from interviews. As recommended when using the CFIR, each memo was subjected to a rating process in order to identify constructs that appear to influence implementation. We used the criteria provided by Damschroder and Lowery to assign ratings to constructs [22]. Each researcher independently assigned a rating to each construct based on valence (positive [+] or negative [−] impact on implementation) and strength (weak [1] or strong [2] impact on implementation) and provided a rationale for the rating. An asterisk (*) was used to identify the existence of a view contrary to the overall rating.

The researchers then created analytic matrices for each CFIR domain that aggregated the entire data set. The matrices included all data included from the analytic memo (i.e., a summary statement for each construct, a rating for each CFIR construct and supporting data from interviews). The three researchers presented the memos for their respective case to each other, and reviewed, deliberated and modified the summary statements and ratings as appropriate. Finally, the researchers produced a narrative summary of patterns of barriers and facilitators across cases, according to CFIR domains. Discussions and feedback among the researchers and the wider team supported reflexivity.

Two researchers, who completed the analysis, identified considerations for service provider organisations when implementing synchronous digital technologies in disability services based on the summary statement for each construct. These were firstly refined through discussion with members of the wider research team. The findings and considerations for implementation were then presented to a wider group of stakeholders that included representatives from service provider organisations and service users, to obtain their interpretation of findings and feedback on considerations for service provider organisations.

## Results

**Fig. 2:**
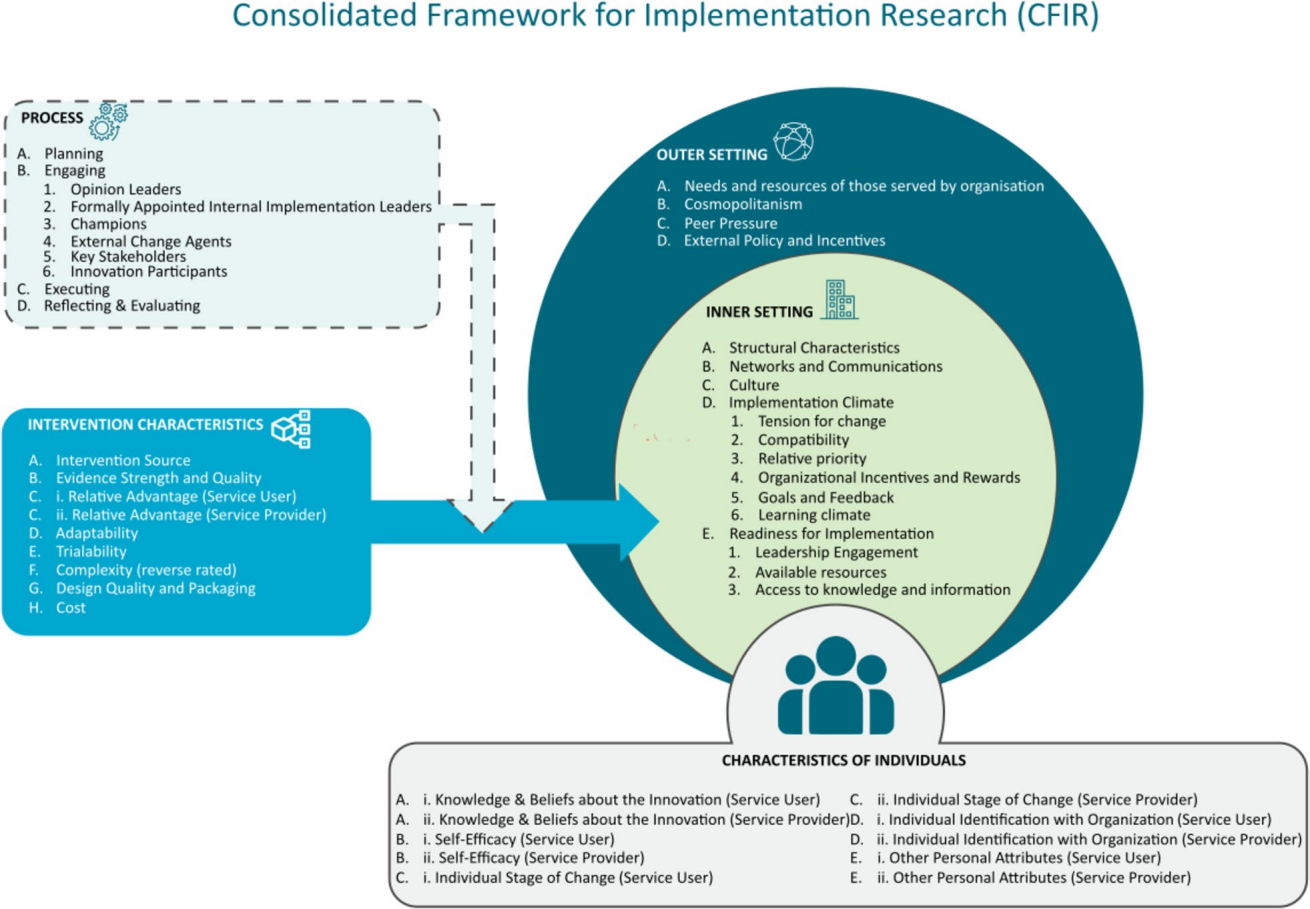
CFIR constructs. Figure adapted from: The Consolidated Framework for Implementation Research (CFIR) 2.0. Adapted from "The updated Consolidated Framework for Implementation Research based on user feedback," by Damschroder, L.J., Reardon, C.M., Widerquist, M.A.O. et al., 2022, *Implementation Sci 17*, 75. Image copyright 2022 by The Center for Implementation. https://thecenterforimplementation.com/toolbox/cfir

Forty interviews were conducted (mean duration 54 minutes; minimum 25 minutes, maximum 113 minutes). We present our findings by CFIR domains. The CFIR constructs within each domain that influenced implementation are described in Fig. 2. Ratings for each construct by case are provided in Table 2. Additional quotes presented by CFIR construct are provided in Additional file 2. In case B, participants used the term “service owner” to describe a person with disability who used the service.

**Table 2:** Ratings assigned to CFIR constructs by case

	**Case A**	**Case B**	**Case C**
**I. INTERVENTION CHARACTERISTICS**			
A. Intervention Source	+2	+2	Missing
B. Evidence Strength and Quality	Missing	Missing	Missing
C. i. Relative Advantage (Service User)	+1*	+2	+1
C. ii. Relative Advantage (Service Provider)	+2*	+2	+1
D. Adaptability	+2*	+2	+2
E. Trialability	Missing	+1	+2
F. Complexity (reverse rated)	-1	-1*	-1*
G. Design Quality and Packaging	-2	-1	-1*
H. Cost	-1	-2	-1
**II. OUTER SETTING**			
A. Needs and resources of those served by organisation	+2	+1*	+2
B. Cosmopolitanism	Missing	+2	+2
C. Peer Pressure	Missing	Missing	Missing
D. External Policy and Incentives	Missing	-1*	-2
**III. INNER SETTING**			
A. Structural Characteristics	Missing	Missing	Missing
B. Networks and Communications	+2	+2*	+2
C. Culture	+1	+1	-1
D. Implementation Climate			
1. Tension for change	+2	+2	+2
2. Compatibility	+1*	-1*	-1
3. Relative priority	-1	-1*	-1*
4. Organizational Incentives and Rewards	Missing	Missing	Missing
5. Goals and Feedback	Missing	Missing	Missing
6. Learning climate	+1	+1*	Missing
E. Readiness for Implementation			
1. Leadership Engagement	+1	+1*	+2
2. Available resources	+1	+2*	+2
3. Access to knowledge and information	+1*	+2*	+1*
**IV. CHARACTERISTICS OF INDIVIDUALS**			
A. i. Knowledge & Beliefs about the Innovation (Service User)	+2*	+2*	+1*
A. ii. Knowledge & Beliefs about the Innovation (Service Provider)	+1	+2*	+1*
B. i. Self-Efficacy (Service User)	+1	+1	+1*
B. ii. Self-Efficacy (Service Provider)	Missing	-1*	+1*
C. i. Individual Stage of Change (Service User)	+1	+2	+2
C. ii. Individual Stage of Change (Service Provider)	+1	+2	+2
D. i. Individual Identification with Organization (Service User)	+2	+2	+2
D. ii. Individual Identification with Organization (Service Provider)	Missing	Missing	Missing
E. i. Other Personal Attributes (Service User)	+2*	+2*	+2*
E. ii. Other Personal Attributes (Service Provider)	Missing	-1*	+2
**V. PROCESS**			
A. Planning	+1	+2	+2
B. Engaging			
1. Opinion Leaders	Missing	Missing	Missing
2. Formally Appointed Internal Implementation Leaders	+2	+2	+2
3. Champions	Missing	Missing	Missing
4. External Change Agents	Missing	+2	Missing
5. Key Stakeholders	Missing	Missing	Missing
6. Innovation Participants	-1*	+1	+1*
C. Executing	+1	+1	+1
D. Reflecting & Evaluating	+1	+2	+1

The valence component of a rating (+, -) was determined by the influence of the coded data on the implementation process

* indicates that the comments were mixed, e.g. a rating of +1* indicates the aggregate of mixed comments was positive

The strength component of a rating (1, 2) was determined by level of agreement among participants, strength of language and use of concrete examples

Missing: interviewees were not asked about the presence or influence of the construct, or if asked about the construct their response was coded to another construct

### Intervention characteristics

Using digital technology to access services remotely had many advantages compared to face-to-face delivery. Advantages included reduced travel, peer support and peer learning, more choice and opportunities to participate, and compatibility with family and/or work commitments. Some also described that accessing supports in their own environment made them feel safer, more comfortable and more like themselves.

“if you then go to the other format which is physical, now a lot more of the available energy you’ve got, has to be spent on logistics and getting there, finding your way. For me personally, let’s get the maximum return on the finite energy we have for this task." Service user (ID11, Case A)

Some people with sight loss stated that the experience of interacting with people online and in person was equivalent, indicating no added benefit to meeting in person.

“So if I’m doing it remotely, I mean I might as well be at the end of a computer because I’m not, I can’t see the person. You know yeah I mean I thoroughly enjoy this remote technology and I hope it continues.” Service user (ID2, Case A).

However, some people preferred to meet in-person because they felt better able to socially connect with others, with this benefit outweighing any challenges they faced travelling.

Service providers also noted advantages to remote delivery compared to face-to-face; their positive perceptions of remote delivery facilitated implementation. Advantages to service providers included reduced travel time, enhanced connection with service users and their families, ability to connect with service users who hadn’t engaged with services previously, and enhanced networking with colleagues nationally. It reduced the time they spent preparing for a session such as setting up a room, checking equipment, and tidying after a session. It also enabled them to connect with service users in advance of in-person meetings to understand their needs, and plan and prioritise topics to cover in-person, which resulted in more efficient in person meetings.

“You literally sit down with the laptop it's much easier, you have the support of admin sending everything out and it's all at the touch of a button there in front of you. There’s more prep that goes into a physical group than an online group, which I wouldn't have thought before doing them both.” Service provider (ID30, Case C)

However, some service providers felt it hindered informal chats and providing individual advice to service users, which they thought could be achieved in person. They also felt less connected with colleagues who did not work directly on the service.

Although no formal pilot was conducted, cases trialed some parts of remote delivery on a small-scale, enabling learning, adaption and wider implementation. The perception that the online services were developed internally, by service providers and sometimes involving service users, strongly facilitated implementation across all cases.

Assistive technology, including iPad mounts, adapted keyboards and screen readers, was essential for enabling people to access online services. Features of the online platform also allowed service providers to adapt the sessions to make them more engaging. However, there were challenges to using assistive technology with the platforms, which caused frustration for some people and even resulted in them giving up on engaging with the online service.

“It’s not a good product in terms of accessibility. It’s quite clunky and cumbersome if you’re relying on voiceover, or other accessibility features. I’ve been using it on various meetings in different groups, over the last eighteen months. So I’ve got to see enough of it to know that it’s not easy. It does present barriers to entry for a lot of people.” Service user (ID11, Case A)

Service users also encountered challenges with audio quality (e.g. interference, echo, poor sound quality for music), video (e.g., limited number of users visible on screen, floating toolbars) and other features (e.g., inability to mute/unmute, difficulties with chat features). Although features were added over time and audio and visual performance improved, these issues negatively impacted the experience for service users and providers.

“I couldn’t unmute myself, because if you keep tabbing around you will eventually get to the mute and unmute button but the problem is that if the trainer is talking in the meantime, it’s very hard to hear the JAWS [screen reader] reading out where you are, when you’re tabbing around” Service user (ID13, Case A)

The complexity of accessing online services and the design of the platforms were barriers to implementation. Multiple steps, such as downloading an app, creating an account, finding the link, using the link, and entering passwords or other information, were needed to join a platform causing frustration for service users.

“they were sending us out links a new link every time and even the staff themselves were getting, not demented but frustrated that they had to send out a new link for something, if it’s the same activity the whole time, they were sending out a new link. But now it’s one link for, is the same link for everything” Service user (ID15, Case B)

Service providers took multiple steps to simplify the process of joining the online service and provided support, which was essential to enable service users to participate.

Costs of implementation included broadband, devices, personnel and assistive technology. For the people we interviewed, cost did not act as a barrier to participating in the online service as they either had the resources required already (e.g. devices, internet) or the organisation provided these. Thus, additional costs were usually borne by the organisation, which facilitated implementation.

### Outer setting

Service providers identified that service users had a need for online services during Covid-19, which facilitated their success.

“We were all busy supporting people on a one-to-one basis. But it was decided look we could support some more people. And it would be a valuable thing to do to have virtual tech clubs.” Service provider (ID10, Case A)

Service users had multiple resources that enabled them to participate, including family or paid support, internet access, having time and flexibility, space and devices. Lack of these resources created challenges to participating in online services.

Linking with external organisations for specialized support, like technical support, facilitated implementation. In some cases, remote service delivery led to the formation of new networks, creating new opportunities for both users and providers.

Despite the identified need for online services, external policies created barriers to implementation. General Data Protection Regulation (GDPR) requirements were considered overly restrictive and created additional complexity for service providers and service users. Further, a national programme to change the way services in Ireland were delivered for children and young people up to 18 years, was being implemented simultaneously and prevented continuity of the online service for children. Finally, external funding supported delivery of the online services but uncertainty about its continuation, and provision of funding based on outcomes, threatened continuity or continuity in its current form.

### Inner setting

The unique context of Covid-19 prompted organisational change, which overcame barriers that may prevent or slow implementation of online services at other times. Managers supported service providers, who felt valued and knowledgeable partners in implementation, and psychologically safe to implement “outside the box” thinking. An existing culture within organisations of valuing partnerships with service users also facilitated implementation. Further, organisations provided the required resources, such as equipment, staff time for providing the service and additional technical support and training.

“just having some protected time in your week for offering these appointments too for that kind of thing. I think it would be good so that it’s seen as important as the other” Service provider (ID31, Case C)

The availability of formal training and information for both service users and service providers was variable, particularly initially. Providing such training would have eased the initial experience for service users and providers. However, service users were very positive about their experience of contacting staff for support. Service providers went above and beyond to support people to join the service. Service user access to this technical support was vital to the success of the online services.

“The people that were running the call were very, very forward in offering help. And accommodating and hopping off the call to try and help someone else get on the call and all that kind of stuff. I’ve noticed with some other groups there’s now a standard long set of instructions that they send out. And I think it’s great to have for people that are new” Service user (ID11, Case A)

Formal and informal communications between service providers, including online meetings, phone calls, emails and online chat, strongly facilitated implementation and created opportunities for local centres to collaborate and establish a national support network. There was also enhanced communication between service providers and service users.

“we’re working more closely now than we were before all of this started because if you were meeting in person or we went on a centre visit we’d only meet the manager once and at least now we can meet all the staff from all over the country once a week or twice a week. We’ve set working relationships with that member of staff. Since we couldn’t meet, this was the best way of reconnecting with people, up and down the country” Service user (ID15, Case B)

However, a small number of service users identified reduced connection with service providers as a result of online delivery.

The online services that were delivered were mostly compatible with an online delivery mode. There were some additional challenges such as facilitating large groups online. However, services and supports that required physical assessment and observation, and one-to-one training or instruction that required practice and feedback, were deemed unsuitable or not optimal for online delivery.

“we would go through a range of exercises. And sometimes we would have to move the computer down or the tablet down to the level so they could see it, in that kind of sense there was a technical difficulty. Because we weren’t at the right angle you know, or we need the feedback.” Parent (ID33, Case C)

Service user choice to access these types of services online was still deemed important while recognising that online delivery may not be the optimal mode of delivery.

Despite successful implementation of online services, there were threats to sustainability. Organisations deprioritised online services as circumstances changed during the Covid-19 pandemic and face-to-face delivery of services returned. Some service users also deprioritised online services as the option for face-to-face delivery returned, but to a lesser degree. Deprioritising online services posed a threat to continuation, despite the relative advantages to service users and the desire among some service users to continue accessing services online. Although hesitancy within organisations to embrace digital technology was overcome to some degree because of the context of Covid-19, it remained a potential barrier to continuing online services.

“I know that other organisations, not health services providers, would have been using Teams on an ongoing basis already. And maybe that’s what’s a bigger problem, there’s a notorious hesitation within the health service to move with the times a little bit and be modern in terms of the digital strategy.” Service provider (ID19, Case C)

Further, lack of awareness of the relative advantages of online services among senior management was perceived as a threat to sustainability. Ongoing resourcing, particularly of support roles, was considered essential for ensuring sustainability.

### Characteristics of individuals

Service users were very positive about online services and valued the connection, interaction and support from service providers and peers. Some service users described personal benefits such as increased confidence, increased self-esteem, and being more vocal. They noted professional benefits including employment opportunities and increased digital literacy.

“It’s amazing, it is really amazing. I’ve had the best 2 years of my life in the pandemic than anybody that I’ve heard of, I’ve flourished” Service user (ID18, Case B)

Service users perceived their organisation as supportive and trustworthy, praising committed staff who went above and beyond in their roles to support them, particularly during the Covid-19 pandemic. Some service users had longstanding relationships with staff, before Covid-19, which contributed to a positive perception of and loyalty to the organisation and staff.

“I felt really, like they’re giving up their time for us, they’re giving up their hours for us and this is amazing and I just felt really emotional towards the whole thing.” Service user (ID18, Case B)

This positive perception meant service users were committed to implementation of online services and wanted to support it in any way they could.

Service providers valued online services, seeing benefits for service users and providers. They believed online services provided a way to connect and impart information, and were valuable to support service users’ mood, mental health and confidence. Staff valued the additional flexibility, increased inclusivity, transferable skills and new approaches to work resulting from online delivery.

“we discovered some things during COVID that were actually brilliant in terms of being able to link with families without having to uproot them from their home, and some scenarios being much more effective by just linking with them online” Service provider (ID29, Case C)

However, some service users had reservations about recording, monitoring and potential privacy and security issues. In addition, parents found supporting their children during sessions stressful due to other commitments. Some service providers shared concerns about privacy and feared that online services would widen inequality by excluding socio-economically disadvantaged service users.

Service users’ self-efficacy in learning how to use technology facilitated engagement. Confidence levels varied and were influenced by age, pre-existing comfort level with technology and pre-existing relationships with others in the online group. It was perceived that those lacking confidence might be willing to take the step to try technology and consequently are difficult to engage

“one of the big challenges with people who are visually impaired is there is this kind of feeling of I could never master that. I feel that there’s an awful lot of people, highly intelligent people and very competent in their own areas that just have some kind of a mental block when it’s a question of getting into technological support for their condition” Service user (ID13, Case A)

However, many service users who expressed low confidence pushed themselves into new situations to access supports for themselves or their children because there was no alternative during Covid-19. Once they overcame the initial fear of trying technology, service users gained experience and became more competent and confident.

“It actually wasn’t that bad, I suppose being over 60, you know and you think turning into my parents, ah Jesus no I can’t handle that, but once you know how to do it its actually not that hard” Service user (ID01, Case A)

As service users became more proficient with technology they could troubleshoot technical issues for themselves and others, which reduced some of the difficulties they initially experienced. As they progressed towards sustained use of the online services, some users led the sessions they had previously participated in.

In addition to self-efficacy, service users’ comfort with group environments or social situations, level of interest in group topics, and previous relationships with other group members facilitated engagement with the virtual services. Problem solving ability, self-awareness of support needs and persistence in overcoming challenges were important characteristics that supported continued engagement.

“I’m quite happy to just roll with it. I might’ve been on it a few times before the technology club. So you know, in that scenario you need to have, I would call it do you know give it a lash and keep going, keep clicking, keep trying ‘til you get there” Service user (ID11, Case A)

Experience with technology supported engagement; however, some service users who did not engage with the online services also described having good familiarity with technology. Personal discomfort with being on camera acted as a barrier to engagement.

Service providers’ self-efficacy in delivering online services impacted implementation. Service providers who believed in their digital capabilities, in the transferability of their skills and who felt comfortable approaching colleagues for support were more confident, which facilitated implementation.

“you get very comfortable very quick. Even though it's online it's your bread and butter as a therapist, you are communicating, you are interacting with children. It's something that I'd be used to from my job so it's just a screen instead of them sitting in front of you. That was the only difference”. Service provider (ID30, Case C)

However, service providers with low digital literacy and limited experience with computers had low confidence and were initially apprehensive about using technology. Through experience, their skills improved and they felt more confident to get involved with delivering the online service and addressing technical issues. They felt that this improved the quality of the service. Service providers’ ability and willingness to act creatively and think outside the box to identify solutions when service users experienced challenges participating in online services also facilitated implementation.

### Process

Services were implemented reactively in response to the COVID-19 pandemic. However, within this reactive response, dynamic planning supported effective implementation. Advance planning among service providers and service users ensured session experience was optimised.

“I created a form to check three different areas. The first area was their level of interest. And following on from that internet access. And how we could respond if they didn’t. Then looking at equipment and supports to organise equipment for them and what kind of support had they at home to support them with logging in. And then what level of training did the family need.” Service provider (ID27, Case B)

This dynamic approach allowed for learning to be shared across networks and shared learning occurred at a more rapid pace than a project implemented under normal circumstances could achieve. In one case, support and resources from an external agency was essential to initiate and grow the service through national roll out.

Service providers possessed a diverse skill-set encompassing the ability to communicate, engage with service users, facilitate groups, and provide technical support. Developing facilitation skills for the online environment was considered a specific skill that was required to successfully implement the online services.

“You’ve got to involve an element of fun to make anything work. And to make it more interesting. Not so much an entertainer, but entertainer in the background, therapist in the foreground. You know thinking on your feet basically. Because you’re seeing this child through a TV screen So kudos to them they did great.” Parent (Case C)

The time and effort that service providers contributed and their championing of the service was essential to its success. Service providers working together to provide support and facilitate the groups further helped implementation.

Various methods were used to advertise online services. Some were systematic and some were not, which risked excluding people who do not typically engage with the organisation. Email invitations were used in some cases, which risked excluding people who lack digital literacy. In all cases, the content of online services evolved in response to input and needs of service users. This happened organically, not always in response to formal feedback, and facilitated successful implementation of the service. All organisations sought formal feedback from service users, though methods differed. Service providers acted on feedback as much as possible to iteratively develop and enhance online services.

## Discussion

Overall, service users and providers were positive about using digital technologies to access and provide disability services and supports remotely. They cited many advantages over in-person delivery including reduced travel time, increased opportunity for peer support and peer learning, more choice and opportunity to participate in activities, and feeling safer and more comfortable in their own home. As a result, the majority of contextual factors that influenced implementation were framed as facilitators to successful implementation. The urgency to identify new modes of service delivery to meet the needs of service users during Covid-19 was a strong facilitator to implementation. However, this was not sufficient for successful implementation. Other factors that were strong facilitators to implementation were the use of adaptations to enable service users to access the online service, the service users’ willingness to try the online service and persistence when they encountered challenges, and the significant time and effort that service providers made to support service users to participate in the online service. However, even in these cases where implementation of digital technologies was successful, service users and providers identified barriers to implementation such as the complexity of accessing online platforms, poor design quality of online platforms, and organisations prioritising in-person delivery over online services.

Key considerations for service provider organisations when implementing synchronous digital technologies in disability services are presented in Table 3. Online delivery offers the opportunity to develop an innovative and distinct programme of services and supports, in collaboration with people with disabilities, that uniquely address the diverse needs of service users. Previous studies reported similar benefits of online services for people with disabilities [11, 17, 24, 25]. In particular, our findings and others [17] highlight the benefit of using technologies to implement services in a group format, which allowed people to develop social connections. In agreement with other studies, we found that using technology for remote service delivery may not be appropriate for some aspects of services such as clinical examination [11, 24]. However, our findings support that service users are given a choice of delivery mode where possible, acknowledging that personal preference, environmental barriers, and competing commitments influence how they choose to access services.

**Table 3:** Considerations for service provider organisations implementing synchronous digital technologies in disability services

**Intervention characteristics**
• Involve people with disabilities and families as equal partners in the process of developing and delivering online services
• Give people with disabilities and families the choice to access all services remotely using technology
• Assess the compatibility of current services with online delivery to identify those suited to online delivery
• Consider developing new online services and supports in a group format that aim to improve knowledge, self-efficacy, advocacy and promote peer-support and connectivity
• Plan for ongoing investment in hardware and software across three phases of acquisition, maintenance and improvement
**Outer setting**
• Regularly monitor the enablers to engagement among people with disabilities and their families, which may include adequate time and space, access to support and devices, access to assistive technology, interest, and confidence
• Share learning from developing and implementing online services for people with disabilities within and between organisations nationally and internationally
• Develop meaningful methods of sharing learning within and between service provider organisations to best meet their needs, which might include guidelines, case studies or instructional videos
• Identify an organisation with responsibility for strengthening existing networks of disability service providers who provide online services, nationally and internationally, to facilitate shared learning
**Inner setting**
• Give service providers dedicated time for planning and collaborative learning, within and between organisations, so that they feel safe and supported to create and trial new online services
• Give service providers dedicated time to develop online services, deliver online services and support online service delivery, distinct to time spent on face-to-face service delivery
• Provide training and information to people with disabilities in accessible forms that meet their diverse needs to enable them to participate in online services as they choose
• Provide training and information to people who support people with disabilities to enable them to assist people with disabilities to engage in online services as they choose
• Co-design training to support delivery of and access to online services with people with disabilities and families
• Resource technical support roles as a distinct and vital role in providing ongoing and individual technical support to people with disabilities, people who support them, and service providers at the point of access
• Support all service providers to access training in basic digital literacy skills
**Characteristics of individuals**
• Provide flexible training approaches to enhance service providers’ technical and online facilitation skills, which are adapted to service providers’ stage of change
• Regularly review the readiness of people with disabilities and families to engage with online services, acknowledging their choice to engage may change over time
**Process**
• Define and resource a “champion” role at all levels of the organisation, to promote sustainability of online services, and support both people with disabilities and service providers to fulfil this role
• Use systematic and inclusive strategies to engage people with disabilities and their families in online services
• Develop and update the content of online services based on the needs of people with disabilities and their families
• Regularly evaluate and actively use data on the number and profile of, and needs of service users engaged in online services, to inform delivery of online services
• Use standardised methods to evaluate the impact of online services

Successful delivery of online services required significant effort from service providers. The ability to adapt the online service to the needs of the individual, reduce the complexity of accessing the online service, and in many cases integrate assistive technologies with digital technologies, was essential to successful implementation. Flexibility from organisations and individual accommodations to address service user needs were previously identified as facilitators to implementing online services for people with disabilities [17, 25]. Similar to a previous study, service providers gathered tips from trialing aspects of online services and used their knowledge of the activity and service user needs to facilitate implementation, rather than undergo training to make the online activities accessible [25]. A firm belief in the organisation’s mission and the value of the services to users contributes to service provider commitment to make online services successful [25].

However, the significant commitment from service providers to enable successful delivery may be unsustainable if the online service is not properly resourced, even when many service users report a strong preference for attending online services over face-to-face services. Similar to previous studies [24, 26], most people in this study had the required devices and connectivity to access online services. However, investment in online services goes beyond acquisition of hardware to include resourcing the acquisition and maintenance of assistive technology, technical expertise and support at the point of access, champions to promote sustainable services, and time for planning and collaborative learning. As such, it is crucial for policy makers and service provider organisations to directly address the requirements of the *Just Digital Framework* [27]: Digital Infrastructure (promoting on-line access); Digital Capabilities (to navigate the digital world); Digital Commodities (access to appropriate hardware and assistive products); and Digital Governance (promoting social inclusion through protecting citizens’ rights, upholding confidentiality, ethical safeguards and security).

Limitations include that data collection was retrospective and we interviewed only five people who did not engage with the service using the video platform; three from case A accessed the service using the telephone and two from case B did not engage at all. Further, the study is limited by the inclusion of only three cases. Although they varied in terms of the people who used the service, the purpose of the service, and the frequency of delivery, they all used a group format and may be described as support services rather than clinical services. Finally, we completed this study prior to the publication of the updated CFIR and therefore did not use the updated constructs [28].

## Conclusion

This study uniquely provides service user and provider perspectives of using synchronous digital technologies to access and provide disability services and supports in Ireland. Structuring our evaluation around the CFIR enabled us to identify facilitators and barriers at multiple levels. The many advantages of online services cited by service users demonstrate why barriers to online service delivery must be addressed. Importantly, the findings indicate that service users should be given the choice to access services remotely acknowledging that personal preference, environmental barriers, and competing commitments influence how they choose to access services. Investment in staff time, technical support, and innovation are essential to enable people with disabilities to participate in online services and supports.

### Supplementary Information

**Additional file 1.** Topic Guide for service users (includes people with disability and support people such as families or personal assistants).

**Additional file 2.** Participant quotes by CFIR construct.

## Data Availability

The datasets generated and analysed during the current study are not publicly available due to our inability to ensure participants will remain anonymous. The datasets used and/or analysed during the current study are available from the corresponding author on reasonable request.

## Abbreviations

UNCRPD: United Nations Convention on the Rights of Persons with Disabilities
NCPPD: National Clinical Programme for People with Disability
CFIR: Consolidated Framework for Implementation Research
GDPR: General Data Protection Regulation
