# Supplementary material for: Understanding the use of digital technologies to provide disability services remotely during the COVID-19 pandemic; a multiple case study design
Source: BMC Health Serv Res. 2024 Mar 11;24:323. doi: 10.1186/s12913-024-10652-6 (PMC10929100; doi:10.1186/s12913-024-10652-6)
Supplement: Supplementary file 1 — Additional file 1. Topic Guide for service users (includes people with disability and support people such as families or personal assistants). [file 12913_2024_10652_MOESM1_ESM.docx]

**Topic Guide for service users (includes people with disability and support people such as families or personal assistants)**

**Can you tell me how you used X service before the pandemic?**

- *Prompts: how long have you being using X service; how frequently did you attend*

**How did you find out that (X service) was going to use (X technology) to provide the service?**

- *Prompts: Choice between technology and alternative service access option; Input in technology choice; Timing of information; Involvement in decision-making; Communication method and format; Discussion opportunities within and outside the organization*

**When you were told that you had to access (X service) via (X technology), how did you feel about it?**

- *Prompts: Prefer face-to-face appointment; Previous experience with X technology; Concerns about using the technology*

**What has been your experience of using (X technology) to access (X service)?**

- *Prompts: Appearance; User-friendly; Able to interact with health professionals and others; Engagement changes over time; Engagement influencing factors*

**Do any issues arise with (X technology) and if so, how do you deal with them?**

- *Prompts: Software updates or technical problems; Who resolves problems; Mechanism for problem resolution*

**Has that the way you use (X technology) in (X service) has changed since it was introduced?**

- *Prompts: Changes in functionality or interface; Switching technology*

**How have you been supported to use (X technology)?**

- *Prompts: Training or support received; Training provider; Mode of training; Opportunity to trial X technology; Ongoing support availability*

**Can you talk about some of the impacts, good or bad, of using (X technology) to access this service?**

- *Prompts:  Impact on the quality of care received; Any costs involved in using X technology for service access (e.g., time, resources, hardware, software); Impact on interaction with the health professionals*

**Is there anything you think could have been done differently to improve your experience of using X technology?**

- *Prompts: Has your feedback changed its use, formally or informally; How has it been adapted to enhance your experience*

**Based on your experience during the pandemic, what are your overall thoughts on using technology to access services remotely?**

- *Prompts: Necessity; Worth the investment; Other realized benefits*

**Would you like to use technology in the future to access (X service)?**

- *Prompts: Would you like to continue to use (X technology)*

**Topic Guide (those responsible for planning implementation)**

**Tell me about your current position in (organisation)?**

**What were your motivations for providing X service remotely?**

- *Prompts: Decision makers; Your role; Internal, external or service user pressure for remote service provision.*

**How did you decide to specifically introduce (X technology) to provide (X service)?**

- *Prompts: Decision makers; Your role; Internal, external or service user pressure for remote service provision; System functionality considerations*

**Once you decided to use X technology, can you describe the process of implementing it in the service?**

- *Prompts: Steps taken; Participants in each step; Start date of the planning process; Teething problems; Changes to the plan; communication method and timing with staff and service users*

**Can you describe your role in this process?**

- *Prompts: Your involvement in steps*

**Looking back at the process you used to initially implement (X technology), what went well?**

- *Prompts: Facilitators; any changes you would make*

**How do you currently use (X technology) in (X service)?**

- *Prompts: Service user demographics; engaged individuals; potential or self-excluded users; staff users*

**Do any issues arise with (X technology) and if so, how do you deal with them?**

- *Prompts: Software updates or technical problems; Who resolves problems; Mechanism for problem resolution*

**I understand that the way you use (X technology) in (X service) has changed since you first introduced it. Can you tell me more about this?**

- *Prompts: Changes in service user demographics; Changes in staff users; Purpose/criteria change; Software, functionality or interface changes How did changes occur? (e.g., within the service or change in implementation strategy/process)*

**What resources were involved in the implementation of X technology?**

- *Prompts: Your time; Others' time; Hardware/Software; Redistribution of resources*

**Were there any barriers to staff using the technology?**

- *Prompts: Improved or worsened over time*

**How do you support staff to use X technology?**

- *Prompts: Training; Hardware; Training provider; Mode of training; Ongoing staff support; New staff support*

**Were there any barriers to service users using the technology?**

- *Prompts: Improved or worsened over time*

**How do you support service users to use (X technology)?**

- *Prompts:* Training; Devices; Training provider; Mode of training; Changes over time; Onboarding new service users

**Can you talk about some of the impacts, good or bad, of introducing (X technology) to deliver this service?**

- *Prompts:* Impact on quality of care provided; Impact on service users; health professionals; the organization

**How have you identified these impacts?**

- *Prompts:* Informal debriefing or feedback; Necessity of evaluation; Staff feedback; Service user feedback

**Based on your experience during the pandemic, what are your overall thoughts on using technology to provide services remotely in your organisation?**

- *Prompts: Necessity; Worth the investment; Other realized benefits*

**How do you think technology in general is going to be used in the future in your organisation?**

- *Prompts:* Future use of X technology in your organization

**Topic Guide for Health Professionals (and other users of the technology to provide services)**

**What is your current role in (organisation)?**

**How did the decision to provide (X service) using (X technology) happen?**

- *Prompts: Decision makers; Your role; Internal, external or service user pressure for remote service provision.*

**Once it was decided to use X technology, can you describe how it was implemented into (X service)?**

- *Prompts: Steps taken; Participants in each step; Timing of information; Teething problems; Changes to the plan; service user involvement*

**Initially, what was your experience of engaging with (X technology)?**

- *Prompts:* Motivation for engaging; Ease or difficulty in engaging with X technology; Dependency of experience based on the service user

**Looking back at the process of how (X technology) was introduced into your service, what went well?**

- *Prompts: Facilitators; any changes you would make*

**How do you currently use (X technology) in (X service)?**

- *Prompts: Service user demographics; engaged individuals; potential or self-excluded users; staff users*

**Do any issues arise with (X technology) and if so, how do you deal with them?**

- *Prompts: Software updates or technical problems; Who resolves problems; Mechanism for problem resolution*

**I understand that the way you use (X technology) in (X service) has changed since you first introduced it. Can you tell me more about this?**

- *Prompts: Purpose/criteria change; Software, functionality or interface changes How did changes occur (e.g., within the service or change in implementation strategy/process)*

**How has your level of engagement with (X technology) changed over time?**

- *Prompts:* What changed; Factors influencing engagement; Reasons for disengagement?

**How engaged are service users?**

- *Prompts:* Varied service user engagement levels initially; Influencing factors; Changes in user engagement change over time?

**How were you supported to use (X technology)?**

- *Prompts: Training; Hardware; Training provider; Mode of training; Ongoing support;*

**How were service users supported to use (X technology)?**

- *Prompts: Training; Devices; Training provider; Mode of training; Changes over time; Onboarding new service users*

**Can you talk about some of the impacts, good or bad, of introducing (X technology) to deliver this service?**

- *Prompts: Comparison with face to face; Impact on the quality of care provided; Added new tasks to your workload; Allowed you to stop any tasks; Changed how you do certain task; Altered interaction with colleagues; Adapt services for provision using X technology; Changes in service user profile*

**How did you identify these impacts?**

- *Prompts:* Organisation feedback mechanism; Informal discussions or formal staff/service user evaluations

**Was there a cost to you or the service involved in using (X technology) to provide (X service)?**

- *Prompts: Your time; Others' time; Hardware/Software; Redistribution of resources; Time/travel saving*

**Based on your experience during the pandemic, what are your overall thoughts on using technology to provide services remotely in your organisation?**

- *Prompts: Necessity; Worth the investment; Other realized benefits*

**How do you think technology in general is going to be used in the future in (your service)?**

- *Prompts:* Future use of X technology in your organization
