# Supplementary material for: Understanding the use of digital technologies to provide disability services remotely during the COVID-19 pandemic; a multiple case study design
Source: BMC Health Serv Res. 2024 Mar 11;24:323. doi: 10.1186/s12913-024-10652-6 (PMC10929100; doi:10.1186/s12913-024-10652-6)
Supplement: Supplementary file 2 — Additional file 2. Participant quotes by CFIR construct. [file 12913_2024_10652_MOESM2_ESM.docx]

**Additional File 2. Participant quotes by CFIR construct**

| **Domain: Intervention characteristics** | |
| --- | --- |
| **Construct** | **Quote** |
| **Domain: Intervention Characteristics** | |
| A. Intervention Source | "one of the tech guys spoke to me about trying to start up a tech club, where you get into little groups and discuss various tech problems and, some of them would be much better than the others and they would help each other. And, so eventually anyway we got this going in our service" Service user (Case A) |
|  | “I got a phone call in April. We’re putting a project together. And we want you involved. I’ve kind of been a guinea pig. Because they were contacting me to see everything was working. And then we set up activities”. Service user (Case B) |
| C i. Relative advantage (service user) | “he didn’t have to get up, get out, go into the car, drive there, wait to go in and then, you know, all that. He just felt he could have his relaxing time because if it was after schoolwork, he could relax for half an hour and he knew he didn’t have to go straight from one thing to the other” Parent (Case C) |
|  | “I’m still in my own surround and I don’t need the transport to go to the centre..and it’s a lot better in my own surroundings. I feel a lot more, safer, isn’t the word. But a lot more myself like I have my own routine. It’s all about choice, but I enjoy it.” Service user (Case B) |
| C ii. Relative advantage (service provider) | "one of the tech guys spoke to me about trying to start up a tech club, where you get into little groups and discuss various tech problems and, some of them would be much better than the others and they would help each other. And, so eventually anyway we got this going in our service" Service user (Case A) |
|  | “I think this has given us the opportunity to, I can sit here now and just talk to a service owner [service user]. Chat away to them you know on a one to one level. Not they’re the service owner and I’m the staff. It’s on a personal level. So for me the big thing is really I have developed relationships with them” Service provider (Case B) |
| D. Adaptability | “They gave me a KVM box. Which is a box where I don’t have to physically touch the laptop. So mute, unmute it’s all through my desktop. They gave me a new keyboard for my desktop, it’s called springboard, so you can press the keys a lot easier. I am typing so much quicker. I don’t know myself; it’s brilliant”. Service user (Case B) |
| E. Trialability | “with the two centres those team were kind of learning with us, as we went along. And like any questions you know, how do we do this, what do you think about that. We’d get the answers and help them. So it was something that really built up as we progressed, you know as we were scaling to more users.” Service provider (Case B) |
|  | “we engaged in a lot of kind of virtual appointments or virtual linking whether that was coaching of families or directly linking with children. And it just kind of grew. So we established this kind of linking-in group” Service provider (Case C) |
| G. Design quality and features | “the limit on the screen was nine. You could have more on the call but they wouldn’t necessarily be visible on the screen. So that’s a limitation…what we try to do is fill the group as much as we could but make sure that we don’t have more than nine people including the  facilitators” Service provider (Case C) |
| H. Cost | “We went up to the highest package because on the low package, it wasn’t enough megabytes for her. And so I went up to the highest package and that helped because it was crashing terrible before that” Service user (Case B) |
|  | “And the financial burden on top of a pandemic and everything must have been very hard for people. I empathise with people that wouldn’t have been able to get that kind of equipment. And from what I understand the service is very good at loaning out tablets to children that didn’t have them” Parent (Case C) |
| **Domain: Outer setting** | |
| A. Needs and resources | “Initially we were making contact by phone calls, just checking in and it became very apparent very fast that they needed more than just a check in once every couple of days. I suppose that’s been developed into looking at a more robust form of alternative service delivery.” Service provider (Case B) |
|  | “I found it strange doing stuff the first lockdown because my house was always full by the time the house emptied it was nearly 5 o’clock and I wasn’t in the humour then to be doing stuff. But now with the laptop I can actually go into my room, put in the head phones and do a meeting as long as the meeting will last” Service user (Case B) |
|  | “I didn’t know how to use it but because my kids were at home, during lockdown so they helped me at that time with a load of stuff. So it was my daughter set everything up and showed me how to use it.” Service user (Case A) |
| B. Cosmopolitanism | “its mad to think I can go from talking to yourself to talking to people outside of the organisation, that I can go wherever I can go virtually. Like it’s amazing to think that that can happen, talking with the people that I do the music with, its amazing” Service user (Case B) |
|  | “Music was really good too. So they had hired an external music therapist”. Service user (Case C) |
| D. External policies and regulations | “The virtual groups pretty much stopped. And that’s mostly because of PDS. The social hangouts group would have absolutely continued and in fact we have gotten questions - could we do another session? Because some of them have moved to another network...from the therapist’s perspective it means you would be providing intervention for another network team, so that’s tricky” Service provider (Case B) |
|  | “it’s just for the passwords and that, that’s the only thing, there’s 3 different sort of log ins but that’s ok, you know that’s the way it is, with GDPR that’s how it is. If it was just a bit, maybe more accessible” Service user (Case B) |
| **Domain: Inner setting** | |
| B. Networks and communication | “I probably talk to some of the other trainers more now. Now we’ve a meeting every Tuesday. And then if I have an issue I’d do a quick teams meeting, or a quick team’s phone call with somebody. You know so I’ve kind of got used to doing that and that works out better for me” Service provider (Case A) |
| C. Culture | “Like if this makes sense there’s no one looking down on you, you’re at the same level as everyone” Service user (Case B) |
|  |  |
| D. Implementation climate;1. tension for change | “I mean without the pandemic we probably wouldn’t have started up the virtual tech clubs” Service user (Case A) |
|  | “I suppose back in 2020 we couldn’t provide anything face to face, in March. So it was the only option to provide a service, so for the majority of caseload it was virtual or nothing” Service provider (Case C) |
| D. Implementation climate; 2. compatibility | “because there was like eighteen people, over twenty. And you could see all the little pictures on the screen. It was nearly impossible to be heard. And I didn’t get any, to say anything because she [facilitator] couldn’t hear me” Service user (Case B) |
| D. Implementation climate; 3. relative priority | “now that Covid, or we’re getting over the fear. It’s every second week is the meeting. And I do miss; it’s a long time, like going from four a month to two a month. And if you weren’t well you’d miss the meeting. And then you know you’d miss the colleagues and the people you’ve got to know” Service user (Case A) |
|  | “More people are kind of getting out and its not being used as much. There is a lot of people still using it and still depending on it which is great but yeah I’d say I’m the one mostly that uses it, in here.” Service user (Case B) |
| E. Readiness for implementation; 1. leadership engagement | “a lot of the decisions we came to in relation to technology clubs and using teams, would be a general meeting among ourselves. At our weekly meeting where people put forward the pros and cons. And we decide as a group, management would be there and they would kind of say, yea that could be a good way to go.” Service provider (Case A) |
|  | “it’s come from the bottom up. So management have to see the differences as well. Not just us, we all see it, on the ground we all see how wonderful it is. It needs to change within the organisation and we need to have people behind us to push it forward” Service provider (Case B) |
| E. Readiness for implementation; 2. Available resources | “They [service] supplied me with an iPad and they’re actually supplying me with a laptop as well. Which is great, I’m actually using the iPad now as I’m talking to you. You know its one way of contacting people. We’d be lost without technology now at this stage, do you know, it’s really brought on an impact” Service user (Case B) |
| E. Readiness for implementation; 3. Access to knowledge and information | “But once we knew how the equipment worked, or what to expect and how to fix it. And it was very helpful to have someone who had done many of these by that point to guide you through.” Parent (Case C) |
| **Domain: Characteristics of individuals** | |
| **Construct** | **Quote** |
| A. i. Knowledge and beliefs about the innovation (service user) | “it was great to have this contact every week during Covid, I really mean that, it has really saved my sanity. Because not alone was it about technology, we gelled, we had the laugh and that was even nicer, you know it wasn’t all learn, learn, learn, learn” Service user (Case A) |
| A. ii. Knowledge and beliefs about the innovation (service provider) | “And it was that transparent window into your home, into someone else’s home. Privacy and appropriateness need to be considered, conversations in the background could be heard, you don't want people giving their personal life away.” Service provider (Case B) |
| B. i. Self-efficacy (service user) | “I definitely see our younger service owners, they’re more au fait with Teams. There’s two new service owners just in service. When I mentioned oh you know we’ve a virtual service and it’s on Teams. They were like, oh yea give me the link, I’ve used it in school. Yea I know it.” Service provider (Case B) |
| B. ii. Self-efficacy (service provider) | “Some staff were very apprehensive about learning. Especially I think a lot of the key workers and that wouldn’t have been computer savvy. I’m not saying not computer literate but learning new stuff online and trying to sort out things for themselves. People aren’t really used to doing that and maybe being a bit afraid” Service provider (Case B) |
| C. i. Individual stage of change (service user) | “some children initially were just watching the group, listening but not taking an active role. And over time they started becoming very vocal and definitely taking part. There was one boy and it was only after maybe three different groups; he started to initiate conversations with peers. And that was just fantastic to see” Service provider (Case C) |
| C. ii. Individual stage of change (service provider) | “This morning I spent an hour dealing with some technical issues, which now I'm a little bit more able to do myself” Service provider (Case B) |
|  | “I think no call is without glitches. Even if there are no glitches from our side, right, I mean some of us I think at this point have become pretty good at ensuring there aren’t any glitches” Service provider (Case C) |
| D. i. Individual identification with organisation (service user) | “the service goes over and above. They do a great job by reaching out to the kids. And we never felt like we were put on the back burner even through the whole crisis. I think that if they can just get props you know for what they do” Parent (Case C) |
| E. i. Other personal attributes (service user) | “she was very adaptable and malleable, that's an even better word. Whatever the situation you show her how to do it and within a couple of tries she can do it herself” Parent (Case C) |
| E. ii. Other personal attributes (service provider) | “A real barrier was, never mind technology itself it was the lack of understanding on how to use it. I mean it's not really on our job description to have high IT skills. Your focus is on new directions and getting people out to the community and that..so IT skills didn’t come into it” Service provider (Case B) |
|  | “I did individual sessions first before I would have done any group sessions. To get really familiar with the technology and all the things that can go wrong. And how you quickly solve it I think is really key. Because the quicker you can iron it out the better” Service provider (Case C) |
| **Domain: Process** | |
| **Construct** | **Quote** |
| A. Planning | “the service spent quite a lot of time establishing what I thought would work, those games, song games. And it kind of, it worked, the more information you get in advance the better you can prepare and hopefully the preparations work”. Service provider (Case C) |
| B. Engaging; 2. formally appointed internal implementation leaders | **“…**we’ll all get a chance to speak. One time there could be a topic, the trainer will go what do you think. And then she’ll go Mark, Luke, John, Paul whatever. And then she might come back the other way in the next topic…and everyone gets to have their say. And I think the chair, or who’s running it is very, it just as important at the meeting as well” Service user (Case A) |
| B. Engaging; 4. external change agents | “we had the partnership with the Microsoft teams. I mean they were great, they volunteered a lot of help with us you know, we had the IT guy. He helped us out a lot as well and so because there was lots of teething problems in the beginning” Service provider (Case B) |
| B. Engaging; 6. Innovation participants | “I found out through another member who to be honest now because I’m in the clique I hear everything but before when I was a member I hadn’t a clue half of the stuff that was going on and was never contacted” Service user (Case A) |
|  | “the craft group got so in demand that we had to do it first come first served response with the children. So like the flyer would go out and then parents would email back” Service provider (Case C) |
| C. Executing | “in the last six months we’ve asked people to come with their own material, with their experience of certain websites, or certain devices..So it’s organically moved in that direction. And I like that. Because it shows the power of the collective learning that we’re doing. That it isn’t just this top down approach” Service user (Case A) |
|  | "the service owners have developed it, kind of nudged it either way. As opposed staff saying oh I think we should do this. It hasn’t been that way at all, it’s been moulded now it’s moulded by the service owners." Service provider (Case B) |
| D. Reflecting and evaluating | “every couple of months we’d send out questionnaires and ask them to fill them up. Where we’d have a number of set tasks, you know questions in relation to the technology club, the virtual session. And then they can feedback whether they liked it or not” Service provider (Case A) |
|  | they’re listening, they call me, “have you some ideas of what we can do?”..We can agree, disagree, I can go to them, no that’s not a good idea. And they go “what if we do this?” compromise it’s great, they're brilliant” Service user (Case B) |
